# Ultrasound Analysis of Respiration-Related Muscles in Rats

**DOI:** 10.3389/fgene.2022.900168

**Published:** 2022-10-28

**Authors:** Shubei Zhan, Bin Zheng, Mengyi Li, Lin Xu, Chengchun Chen, Peizhen Huang

**Affiliations:** ^1^ Department of Ultrasound and Imaging, Wenzhou Central Hospital, Wenzhou, China; ^2^ Wenzhou Medical University, Wenzhou, China

**Keywords:** ultrasound, diaphragm, tidal volume, rapid shallow breathing index, rectus abdominis ultrasound imaging, a non-invasive technique for assessing organ function, has been used

## Abstract

The purpose of this study was to evaluate the effectiveness of ultrasound techniques in the analysis of respiratory-related muscles in rats. Respiratory parameters, including diaphragm end-expiratory thickness, mean rectus abdominis (RA) thickness, and RA area, were measured by ultrasound and compared with histological findings. Spearman’s correlation and Logistic regression analysis were used to detect the differences in the correlation between ultrasound results and histological examinations, and Student’s t test was used to compare the differences between ultrasound results and histological examination data. The results showed that there was no significant difference between the end-expiratory thickness of the diaphragm, the average thickness of RA, and the area of RA in the right RA and histological values under ultrasound detection (*p* > 0.05), but there was a significant positive correlation between ultrasound, and histological values (*p* < 0.05).); in addition, tidal volume was significantly positively correlated with total RA area, rapid shallow breathing index (RSBI) was significantly negatively correlated with total RA area, and mean diaphragm TF was significantly positively correlated with tidal volume. In conclusion, ultrasound imaging has a high degree of accuracy and reproducibility and can be used to assess the structure and function of the rat diaphragm and RA.

## Introduction

Ultrasound imaging, a non-invasive technique for assessing organ function, has been used mostly to assess left ventricular systolic function in animal studies but is less common in other organs (Chen et al., 2021; Zang et al., 2021). Lung and respiratory muscle function in animals is often assessed indirectly by plethysmography, mostly used to assess respiratory dysfunction following neuromuscular disease or cervical spinal cord injury (De Troyer et al., 1989; Farkas et al., 1994). In addition, the animal’s respiratory muscle function can be recorded and analyzed by diaphragm electromyography under anesthesia or using telemetry implants in the awake state. However, these techniques are invasive, do not provide longitudinal studies, and require animal sacrifice (Fuller et al., 2008; Fayssoil and Tournoux, 2013). Therefore, the development of non-invasive tools for assessing respiratory function in animal models is necessary and will facilitate the evaluation of potential drugs for therapeutic applications. Ultrasound has been used to assess the respiratory muscles in humans, and the diaphragm is considered the primary inspiratory muscle. In preclinical models, multiple studies have demonstrated the efficacy of ultrasound to assess diaphragm function in canine muscle disease models and in mouse models of neuromuscular disorders (Goyenvalle et al., 2015; Jeong et al., 2017). However, expiratory muscles are also actively involved in respiratory behavior, including the rectus abdominis (RA), external oblique (EO), internal oblique (IO), and transverse abdominis (TA), especially in rodents and dogs. In the setting of severe diseases such as spinal cord injury, the functional activity and efficacy of these breathing-related muscles may be affected (Kobayashi et al., 2001; Laghi and Tobin, 2003; Lee et al., 2014). To date, there are very few data on the assessment of respiration-related muscles by ultrasound in a rat model, whereas this study used ultrasound to describe and evaluate the physiological values of diaphragm muscle (thickness, thickening, and inspiratory motion) and RA muscle thickness in anesthetized rats, and these data can provide a reference for ultrasound measurements of the diaphragm and RA in rats at steady state.

## Methods

### Animal

Fifteen adult male Sprague Dawley rats (291–300 g, 8 weeks old, Laboratory animal license number: SCXK (Su) 2019-0005, Animal ethics approval number: ethics approval KZ20210416) were purchased from Beijing Life River Animal Experiment Co., Ltd. and acclimated to a constant temperature (22°C) and 12–12 h light-dark cycle for 7 days before proceeding with subsequent experiments. During the experiment, the rats had ad libitum access to food and water.

### Experimental Program

Measurement of different parameters under anesthesia by ultrasound (5% isoflurane followed by 2% isoflurane to maintain anesthesia): end-expiratory and end-inspiratory left and right diaphragmatic muscle thickness during spontaneous ventilation in animals at rest, the thickness and area of the left and right RA during spontaneous ventilation in the resting state, and the thickness and area of the left and right diaphragms of the same rat were also studied by plethysmography and histology.

### Whole Body Plethysmography

Assess the animal’s overall ventilatory function (e.g., tidal volume, minute ventilation, and respiratory rate) by constant flow (0.5 L/min) plethysmography: calibrate the system and weigh the rats, Start recording under normoxic conditions after a 30-min acclimation period in the chamber, tidal volume and minute ventilation were reported as the body weight of each animal (per 100 g), and the Rapid Shallow Breathing Index (RSBI) was calculated by respiratory rate [RR]/tidal volume.

### Ultrasound of the Diaphragm and Rectus Abdominis

Respiratory muscle function was assessed at high frequency (18 MHz) using a high-resolution ultrasound system and a linear L8-18i probe. All animals were anesthetized with 5% isoflurane at a rate of 500 ml/min, and the isoflurane concentration was subsequently reduced to 1.5–2% after anesthesia to maintain anesthesia. Use a razor and depilatory cream to remove the animal’s prothorax and epigastric hair, place the animal in a supine position on the imaging platform, monitor body temperature continuously using a rectal probe and maintain it at 37 ± 0.5°C, and ultrasound gel was applied to the thorax of the rat, and M-mode ultrasound was used to assess diaphragm muscle movement from a subcostal view.

Diaphragm thickness and thickening of the diaphragmatic apposition area during live breathing were assessed using 2D mode: the probe was placed perpendicular to the chest wall at the midaxillary line to visualize the costophrenic sinus, images of the diaphragm were recorded, and the thickness of the diaphragm at the end of expiration and inspiration was recorded, the fractional thickening (TF) of the diaphragm was calculated: ([end-inspiratory thickness]-[end-expiratory thickness])/[end-expiratory thickness] (Lim et al., 2014).

RA analysis: The probe is placed vertically in the upper abdomen, just above the umbilicus, then moved to the middle white line, the right and left RA muscles are recorded (the white line is surrounded by a muscle sheath on each side), and thickness and area are measured.

### Texture Analysis

After the rats were sacrificed by cervical dislocation, the diaphragm and RA were dissected and transverse paraffin sections (7 μm thick) were made. After dehydration treatment, hematoxylin-eosin staining was performed. After scanning the slides, ImageJ software was used for analysis. Measure the thickness and area of the diaphragm and RA, and the thickness of the diaphragm was taken as the average value of five different positions in the same slice, and the thickness of RA was taken as the measured thickness at the maximum position of the same slice.

### Statistical Analysis

Data were presented as mean ± SD, statistical significance was defined as *p* < 0.05, and the correlation between ultrasound results and histological examination was tested by Spearman’s correlation and logistic regression analysis, and Student’s t test was performed to compare ultrasound results with histological examination, within-observer (A1vs..A2) and between-observer (A1vsB1) variability were analyzed by Bland-Altman plots, and if the results were within the 95% concordance limit, the agreement was good, and all data were analyzed by SPASS 20.0.

## Results

### Whole-Body Plethysmography in Unanesthetized Rats

A total of 15 rats were included in this study. Inspiratory time (178 ± 29 ms), expiratory time (376 ± 101 ms), minute ventilation (39.6 ± 11.2 ml/min/100 g), tidal volume (0.38 ± 0.12 ml/100 g), respiratory rate (119 ± 26 breaths/min), and rapid shallow breathing index (278 ± 45, Table 1).

**TABLE 1:** Whole body plethysmography in unanesthetized rats.

Variables	Mean ± SD	Kappa
weight (g)	315 ± 63	
TI (ms)	178 ± 29	20
TE (ms)	376 ± 101	34
VM (ml/min)/100 g	39.6 ± 11.2	8.95
VT (ml)/100 g	0.38 ± 0.12	23.90
Respiratory rate (times/min)	119 ± 26	21
RSBI (RR/VT)	278 ± 45	24

Notes: Inspiratory Time (TI), Expiratory Time (TE), Minute Ventilation (VM), Tidal Volume (VT), RSBI (Rapid Shallow Breathing Index), Minimum (min) and Maximum (max), Sample size(*n*) = 15.

### Intraobserver Repeatability and Interobserver Variability of Ultrasound Measurements

After the sonographer measured the relevant data (RA area, RA thickness, diaphragm thickness, and end-inspiratory diaphragm thickness) twice, the Bland-Atlman method showed that all data points were within the 95% consistency limit, the results have good consistency. After different sonographers measured relevant data (RA area, RA thickness, diaphragm thickness, and end-inspiratory diaphragm thickness), the Bland-Atlman method showed that 4.83% of the data points were outside the 95% limit of agreement, within the limits of consistency, the maximum difference between the two (the area of RA on the right side) was 2.13 mm^2^, and the results were in good agreement.

### Ultrasound and Histology Results

The end-tidal thickness of the right diaphragm in anesthetized rats (0.76 ± 0.05 vs. 0.82 ± 0.04 mm), the end-tidal thickness of the left diaphragm (0.79 ± 0.08 vs. 0.85 ± 0.07 mm), the average thickness of the right RA (1.69 ± 0.18 vs. 2.17 ± 0.32 mm), left RA mean thickness (1.74 ± 0.15 vs. 2.08 ± 0.27 mm), right RA area (22.8 ± 1.52 vs. 21.9 ± 1.18 mm^2^), left RA area (23.4 ± 1.43 vs. 21.8 ± 0.96 mm^2^), there was no significant difference between the ultrasound and histological values, and there was a clear positive correlation between the ultrasound and histological values (Table 2).

**TABLE 2:** Concordance and correlation between ultrasound and histological measurements.

变量	Ultrasound	Histological	X^2^	*p*	R	*p*
End-expiratory thickness of right diaphragmatic membrane (mm)	0.76 ± 0.05	0.82 ± 0.04	1.258	0.792	0.751	0.009
End-expiratory thickness of left diaphragmatic membrane (mm)	0.79 ± 0.08	0.85 ± 0.07	1.362	0.683	0.816	0.002
Right RA thickness (mm)	1.69 ± 0.18	2.17 ± 0.32	2.186	0.219	0.782	0.027
Left RA thickness (mm)	1.74 ± 0.15	2.08 ± 0.27	2.132	0.376	0.569	0.021
Right RA area (mm^2^)	22.8 ± 1.52	21.9 ± 1.18	0.815	0.925	0.812	0.001
Left RA area (mm^2^)	23.4 ± 1.43	21.8 ± 0.96	0.996	0.867	0.723	0.001

### Ultrasound Results of Diaphragm TF

Ultrasound results showed that the fractional thickening of the diaphragm was similar on both sides: the TF of the right diaphragm was 24.6 ± 5.83%, and the TF of the left diaphragm was 23.8 ± 6.02%, and the inspiratory motion of the diaphragm was also similar: The end-expiratory thickness of the right diaphragm was 3.92 ± 0.78 mm, and the end-expiratory thickness of the left diaphragm was 3.88 ± 0.86 mm.

### Relationship Between Rectus Abdominis Area, Diaphragm and Tidal Volume and Rapid Shallow Breathing Index

**FIGURE 1:**
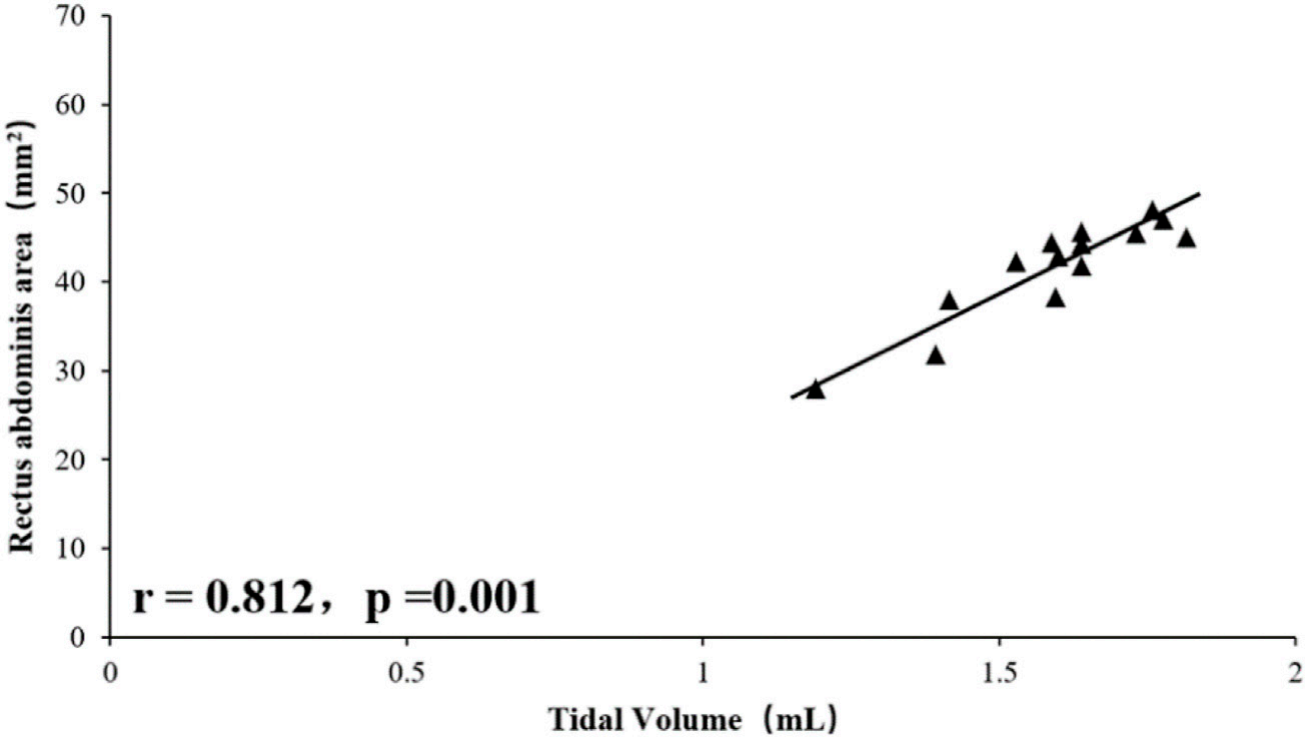
Relationship between tidal volume and rectus abdominis area.

**FIGURE 2:**
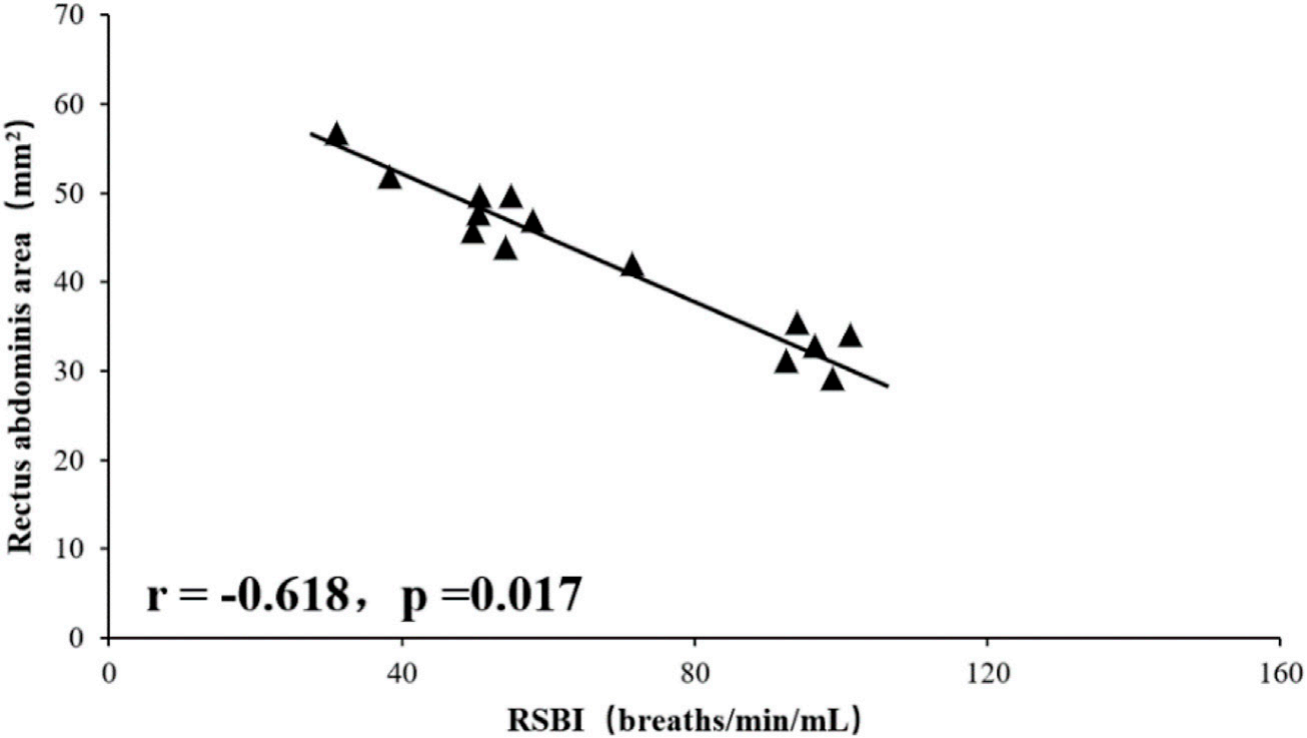
Relationship between BRSBI and rectus abdominis area.

**FIGURE 3:**
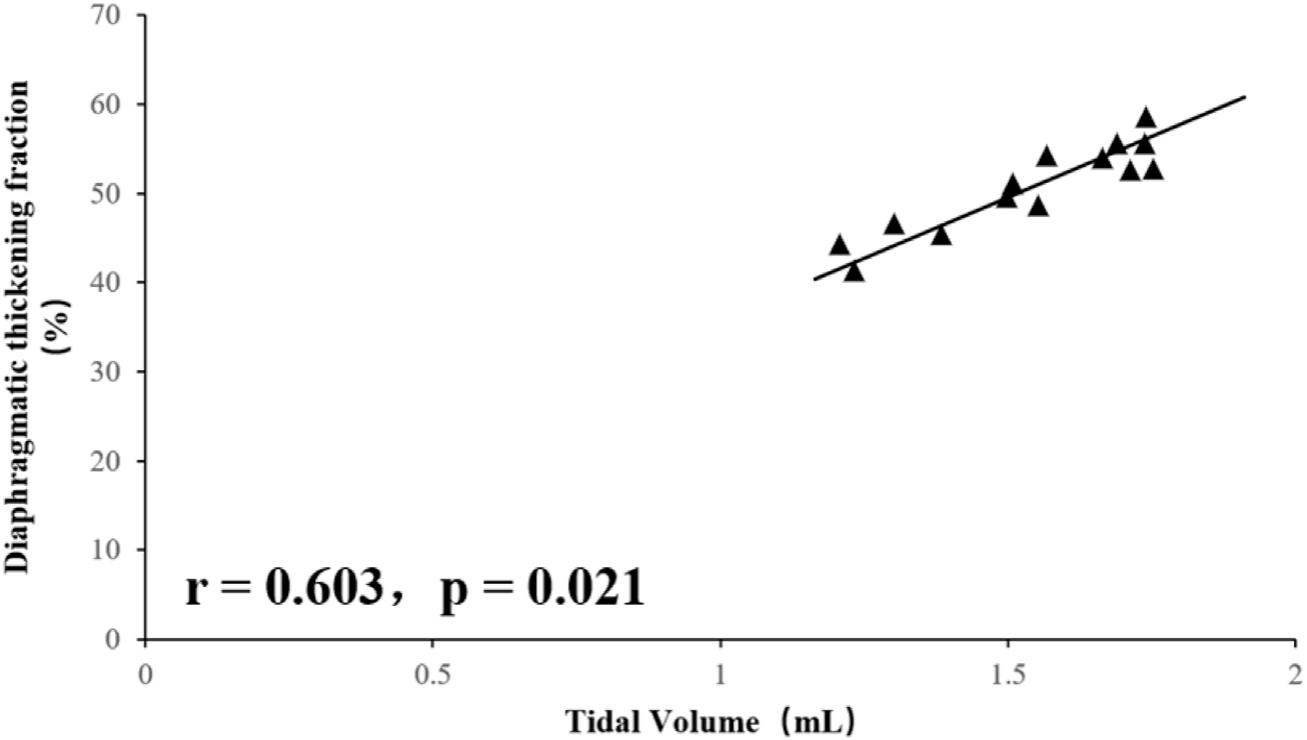
Relationship between Tidal Volume and Diaphragm TF.

**FIGURE 4:**
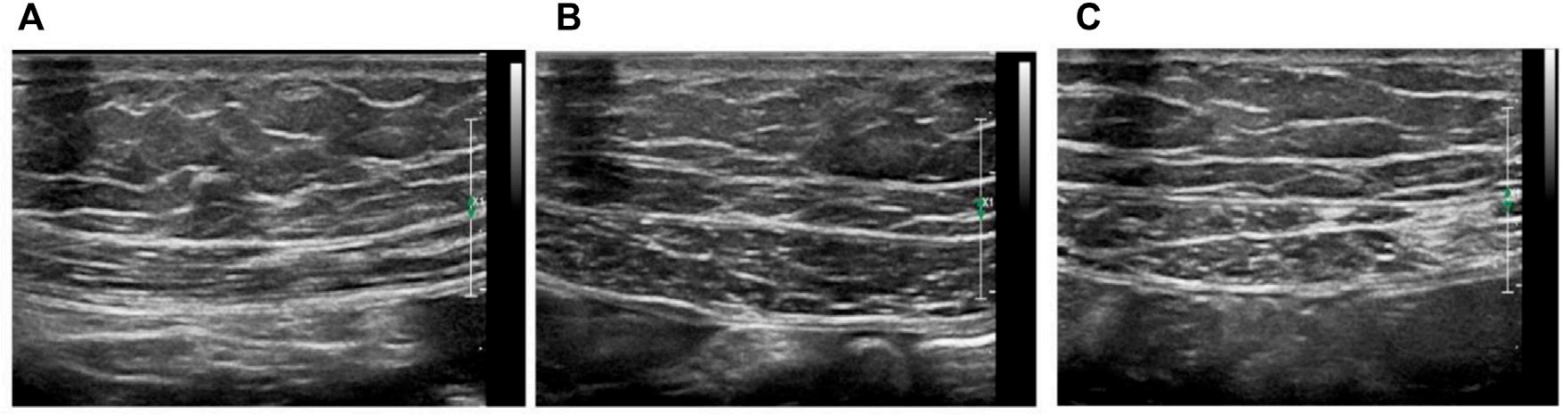
Ultrasound analysis of rat respiratory muscles **(A)** Ultrasound measurement of diaphragm thickness, **(B)** Ultrasound images of left and right **(C)** rectus abdominis, bar = 2 mm.

Tidal volume was significantly positively correlated with total RA area (right RA total + left RA total area) (r = 0.812, *p* = 0.001), and RSBI was significantly negatively correlated with RA total area (r = −0.618, *p* = 0.017), the mean diaphragm TF ((left diaphragm TF + right diaphragm TF)/2) was significantly positively correlated with tidal volume (r = 0.603, *p* = 0.021, Figures 1–4), while the mean diaphragm exhalation End-expiratory thickness ((left-end-expiratory thickness of left diaphragm + right-end-expiratory thickness of right diaphragm)/2) was not associated with tidal volume (*p* = 0.361), and RSBI index was negatively correlated with mean end-expiratory thickness of diaphragm, but not significantly (r = −0.251, *p* = 0.129).

## Discuss

This study is the first to use ultrasound to detect diaphragm, RA thickness and area data in rats, obtained by non-invasive ultrasound techniques, and validated by histological analysis. The results confirm that in a rat model, experimental data support the high reproducibility of this technique for assessing diaphragm and RA muscle assessments.

A total of 15 rats were included in this study, and the values of inspiratory time, expiratory time, tidal volume, minute ventilation, and respiratory rate for steady breathing in unanesthetized rats were similar to those reported in the literature (Lim et al., 2014). Ultrasound has previously been used to assess diaphragm function in mice and found a significant correlation between diaphragmatic inspiratory movements and *ex vivo* values (histological findings) (Lovett-Barr et al., 2012; Lim et al., 2014). Consistent with this, in this study, the results showed a positive correlation and consistency of the data related to the diaphragm or RA with the histological findings. In humans, Wait (Wait et al., 1999) in 1989 reported ultrasonographic data on diaphragm thickness, which were confirmed by autopsy studies to be highly correlated (r = 0.93, *p* < 0.001). In the present study, using a high-resolution frequency ultrasound probe, a similar correlation was found between the rat ultrasound and histological data, and the deviation of the rat ultrasound and histological data can be determined by hematoxylin-eosin staining previous histological processing to explain, because unfixed muscle tissue sections must be dehydrated before staining. On the other hand, the tactile pressure of the ultrasound probe in use can artificially reduce the size of the tissue; furthermore, the animal is alive, so the muscles are not fully relaxed even when their size is analyzed when they are relaxed. Thus, the values obtained by ultrasound techniques are close to histological values and true physiological measurements, and the results of this study demonstrate the usefulness of ultrasound imaging in assessing respiratory muscles in living animals. Correlation analysis showed that tidal volume measured in unanesthetized rats was significantly associated with RA area and fractional diaphragmatic thickening, a finding that suggests that both the diaphragm, and RA are involved in rat breathing. Probably because the RA is located in a ventral location in rats and is responsible for the maintenance of visceral and intra-abdominal pressure in the abdominal cavity. In humans, expiratory muscle thickness increases significantly during forced exhalation, including RA, TA, and IO, and the thickness of TA is significantly associated with gastric pressure generated during forced exhalation (Wen et al., 2019). In animals, De Troyer et al. demonstrated by electromyography (De Troyer et al., 1989) that the TA is the main expiratory muscle in dogs during steady breathing. In the present study, RA was involved in the maintenance of tidal volume in rats, as evidenced by the positive correlation between RA area and tidal volume. In addition, rats with high RA area showed reduced respiratory load and manifested as a significant negative correlation between RSBI and RA area. This finding also suggests that RA is required for the rat’s respiratory cycle.

Analysis of respiratory function is critical and needs to be explored using relevant parameters close to *in vivo* conditions. In animals, this analysis is limited to *in vivo* recordings from invasive EMG and telemetry implants, or indirect data from plethysmography. These techniques are often invasive, do not provide longitudinal studies, and require animal sacrifice. The development of non-invasive tools for assessing respiratory function in rodent models is necessary and will facilitate the evaluation of drugs for potential therapeutic applications. This study demonstrates the feasibility and relevance of ultrasound imaging to study respiratory parameters in rats, and provides a reference for future research. However, this study also has certain limitations, because of the feasibility of obtaining imaging, the study focused on the diaphragm and RA, and did not include other abdominal muscles: EO, IO, and TA.

## Data Availability

The original contributions presented in the study are included in the article/Supplementary Material, further inquiries can be directed to the corresponding authors.

## References

[B1] Chen X. Zhang J. Wang C. (2021). Establishment of a Mouse Model of Heart Failure with Preserved Ejection Fraction [J]. J. Shanghai Jiaotong Univ. (Medical Edition) 41 (05), 565–570. 10.3969/j.issn.1674-8115.2021.05.002

[B2] De Troyer A. Gilmartin J. J. Ninane V. (1989). Abdominal Muscle Use During Breathing in Unanesthetized Dogs. J. Appl. Physiol. (1985) 66 (13), 20–27. 10.1152/jappl.1989.66.1.20 2521846

[B3] Farkas G. A. Gosselin L. E. Zhan W. Z. Schlenker E. H. Sieck G. C. (1994). Histochemical and Mechanical Properties of Diaphragm Muscle in Morbidly Obese Zucker Rats. J. Appl. Physiol. (1985) 77 (126), 2250–2259. 10.1152/jappl.1994.77.5.225010.1152/jappl.1994.77.5.2250 7868442

[B4] Fayssoil A. Tournoux F. (2013). Analyzing Left Ventricular Function in Mice with Doppler Echocardiography. Heart Fail. Rev. 18 (316), 511–516. 10.1007/s10741-012-9345-8 22961495

[B5] Fuller D. D. Doperalski N. J. Dougherty B. J. Sandhu M. S. Bolser D. C. Reier P. J. (2008). Modest Spontaneous Recovery of Ventilation Following Chronic High Cervical Hemisection in Rats. Exp. Neurol. 211 (39), 97–106. 10.1016/j.expneurol.2008.01.013 18308305 PMC2613014

[B6] Goyenvalle A. Griffith G. Babbs A. El Andaloussi S. Ezzat K. Avril A. (2015). Functional Correction in Mouse Models of Muscular Dystrophy Using Exon-Skipping Tricyclo-DNA Oligomers. Nat. Med. 21 (68), 270–275. 10.1038/nm.3765 25642938

[B7] Jeong W. S. Lee S. S. Park E. J. Han J. J. Choi J. W. Koh K. S. (2017). Comparison of Biomechanical and Histological Outcomes of Different Suture Techniques in Rat Rectus Abdominis Muscle Repair. Ann. Plast. Surg. 78 (15), 78–82. 10.1097/SAP.0000000000000800 27070676

[B8] Kobayashi K. Lemke R. P. Greer J. J. (2001). Ultrasound Measurements of Fetal Breathing Movements in the Rat. J. Appl. Physiol. (1985) 91 (136), 316–320. 10.1152/jappl.2001.91.1.316 11408446

[B9] Laghi F. Tobin M. J. (2003). Disorders of the Respiratory Muscles. Am. J. Respir. Crit. Care Med. 168 (11), 10–48. 10.1164/rccm.2206020 12826594

[B10] Lee K. Z. Huang Y. J. Tsai I. L. (2014). Respiratory Motor Outputs Following Unilateral Midcervical Spinal Cord Injury in the Adult Rat. J. Appl. Physiol. (1985) 116 (37), 395–405. 10.1152/japplphysiol.01001.2013 24285148

[B11] Lim R. Zavou M. J. Milton P. L. Chan S. T. Tan J. L. Dickinson H. (2014). Measuring Respiratory Function in Mice Using Unrestrained Whole-Body Plethysmography. J. Vis. Exp. 32 (198), e51755–3600. 10.3791/5175510.3791/51755 PMC4827935 25146417

[B12] Lovett-Barr M. R. Satriotomo I. Muir G. D. Wilkerson J. E. Hoffman M. S. Vinit S. (2012). Repetitive Intermittent Hypoxia Induces Respiratory and Somatic Motor Recovery after Chronic Cervical Spinal Injury. J. Neurosci. 32 (158), 3591–3600. 10.1523/JNEUROSCI.2908-11.2012 22423083 PMC3349282

[B13] Wait J. L. Nahormek P. A. Yost W. T. Rochester D. P. (1999). Diaphragmatic Thickness-Lung Volume Relationship *In Vivo* . J. Appl. Physiol. (1985) 67 (412), 1560–1568. 10.1152/jappl.1989.67.4.1560 2676955

[B14] Wen M. H. Wu M. J. Vinit S. Lee K. Z. (2019). Modulation of Serotonin and Adenosine 2A Receptors on Intermittent Hypoxia-Induced Respiratory Recovery Following Mid-Cervical Contusion in the Rat. J. Neurotrauma 36 (198), 2991–3004. 10.1089/neu.2018.6371 31099299

[B15] Zang M. L. Yu M. D. Chen Z. H. Huang M. Q. Luo P. Fan H. K. (2021). Dynamic Changes of Cardiac Structure and Function in Mice with Abdominal Aortic Coarctation [J/OL]. Chin. J. Appl. Physiol. 07 (27), 1–4. 10.12047/j.cjap.6113.2021.065 34816656

